# Fuzzy Comprehensive Evaluation Model of M&A Synergy Based on Transfer Learning Graph Neural Network

**DOI:** 10.1155/2021/6516722

**Published:** 2021-10-11

**Authors:** Mingxun Zhu, Zhigang Meng

**Affiliations:** ^1^School of Economics and Management, Changsha Normal University, Changsha 410100, China; ^2^School of Computer Engineering and Applied Mathematics, Changsha University, Hunan, Changsha 410003, China

## Abstract

With the rapid development of modern China and the influx of capital, the number of companies has gradually increased. However, most companies cannot operate for a long time due to various reasons. Therefore, mergers and acquisitions have occurred. Large companies merge small companies to some extent. The number of employees can be guaranteed, and the market can be stabilized. However, mergers and acquisitions also have higher risks. As the pace of mergers and acquisitions accelerates, there are more and more cases of failed mergers and acquisitions. The synergy effect of mergers and acquisitions is an important indicator to judge the performance of mergers and acquisitions. This article measures the synergy obtained by the main enterprise from the perspective of performance changes, establishes an evaluation model through the rate of change of financial indicators and migration learning, estimates it through a neural network model, and conducts an empirical analysis on it. The transfer learning neural network has been studied in depth. The research of this article is to accurately assess the synergy effect obtained after mergers and acquisitions and to analyze whether the company can profit from mergers and acquisitions, so as to provide a reference for subsequent mergers and acquisitions between companies.

## 1. Introduction

Mergers and acquisitions of enterprises are the products of the market economy. They generally refer to a property rights transaction in which a company obtains some or all of the property rights of other companies under the action of the market mechanism to obtain the control of the company.

After the market economy develops to a certain stage, it needs the concentration of production and capital, that is, to further integrate resources through mergers and acquisitions to achieve long-term growth of the corporate value. It can be said that corporate mergers and acquisitions are a process of resource allocation and redistribution. Since the century, there have been five waves of large-scale mergers and acquisitions worldwide. For many companies, growth is the basic condition for their survival and development, and corporate mergers and acquisitions are the main way for companies to expand outside.

The development of mergers and acquisitions in China is relatively late. From then on, China began to accelerate the development of mergers and acquisitions in various forms. According to incomplete statistics, in the year of merger, a total of listed companies with shares initiated a reorganization plan, accounting for the total number of listed companies. From year to year, China's corporate mergers and acquisitions and reorganizations occurred more than one year, and the amount involved was as high as 100 million yuan. China's listed companies, the mergers and acquisitions that occurred at this stage are related to the acquisition of shell resources and are financial mergers and acquisitions with the goal of seeking short-term profit growth and value transfer.

With the basic completion of the share reform of listed companies in my country in 2009, the problem of the same share and the same rights has been solved, and my country's stock market has entered the era of full circulation. When shares can flow, their role as a payment tool will emerge, which breaks through the constraint of relying on cash as a payment tool in the case of equity split. The new “Company Law”, “Securities Law”, and “Administrative Measures for the Acquisition of Listed Companies” were successively promulgated, which formulated new rules for the M&A market in the era of full circulation, greatly enhanced the operability of mergers and acquisitions of listed companies, and provided a better payment method for mergers and acquisitions. Diversification solves institutional barriers.

This article is divided into five parts: the first part is the research background, the second part is the literature review, analyzing the research results of the problem; the third part is the introduction of the fuzzy comprehensive evaluation model of merger synergy based on the transfer learning neural network and the introduction of related systems; the fourth part is specific experimental analysis, demonstrating the evaluation effect of the fuzzy comprehensive evaluation model of merger synergy based on the transfer learning neural network and comparing it with the traditional method; and the fifth part is the conclusion of the article.

### 1.1. The Related Works

The event research method is based on the hypothesis of efficient market theory, treats corporate M&A transactions as a single event, and evaluates M&A performance by analyzing the fluctuations in the secondary stock market of both parties within a certain period of time before and after the announcement of the M&A event to reflect the impact of this event. [1]. The event research method usually uses the abnormal return of stocks to measure the stock price fluctuations. It compares the actual return in a certain period of time before and after the release with the normal return in the same time under the assumption that the event does not occur, and the abnormal return is obtained when extraordinary income within the range is accumulated called the accumulative unconventional income. The accumulated unconventional income situation can reflect the synergistic effect of mergers and acquisitions. Because scholars can choose the length of the research interval to analyze the short-term or long-term impact of mergers and acquisitions on the capital market, respectively, the event research method has become one of the most commonly used methods for testing the synergy of mergers and acquisitions. Although there are differences in the selection of samples, test intervals, and merger methods, existing studies have basically reached a unified conclusion on the changes in the wealth of target company shareholders. The target company shareholders can always obtain statistically significant cumulative average abnormal returns. That is, the shareholders of the target company can always make profits in M&A transactions, and only the size of the benefits is different [2–5]. Taking a case of mergers and acquisitions that occurred during the year as a research sample for empirical analysis, it is found that, during the event period , the shareholders of the target company have obtained them. In addition, on the basis of the relevant literature in the summary article, it is pointed out that on successful mergers and acquisitions, if the target company is merged, shareholders can enjoy the excess remuneration, while the shareholders of the merging enterprise cannot enjoy significant excess remuneration; if the takeover method is adopted, the shareholders of the target enterprise enjoy the excess remuneration, while the shareholders of the merging enterprise only enjoy the excess remuneration. By studying a merger event during the year later, the author found the behavior of the target company's shareholders in the event window. After a comprehensive analysis of the documents of the years, it is concluded that the target company's shareholders' income in the mature market M&A process is much higher than the M&A's shareholders' income.

Chinese scholars use this method to study the effect of mergers and acquisitions, and their conclusions are not the same. Chen Xinyuan and Zhang Tianyu conducted research on the mergers and acquisitions activities of listed companies in the same year and found that from the day before the announcement of the merger to the days after the announcement, although the main merged company has an upward trend, the statistical results are not significantly different. Dai *Z* et al. collected the mergers and acquisitions of listed companies in Shanghai and Shenzhen from year to year. Research shows that through mergers and acquisitions, the value of the target company has increased, while the value of the merger and acquisition company has remained basically unchanged. In other words, the market believes that in the current M&A transactions, the target company can gain value from it, and the acquirer will not benefit from the merger [6]. When Xia P et al. studied the case of a share swap and merger between Tsinghua Tongfang and Lu Ying Electronics during the research year, it was found that Tsinghua Tongfang's shareholders and Lu Ying Electronics' shareholders received approximately positive cumulative abnormal returns during the window period. The positive cumulative abnormal return rate was about 10 million yuan, and the merger also produced a positive synergistic effect of about 100 million yuan [7]. In this regard, Hou B et al. hold different views. Through empirical research on the total mergers and acquisitions in Shenzhen and Shanghai from year to year, they found that the shareholders of the main merger company obtained positive cumulative abnormal returns in the interval from the first closing of the announcement to the second closing of the announcement. At the same time, the target company failed to obtain significant accumulated abnormal returns. That is to say, mergers and acquisitions can bring a significant increase in the wealth of the main merged company, while the impact on the wealth of the target company's shareholders is not significant. The advantage of this method is that research data can be easily obtained in the open market, but it is difficult to grasp the event interval, and the sample of listed companies that must be obtained in the open and effective stock market has research value [8]. The shorter the time, it is difficult for researchers to systematically estimate all the information and impacts related to mergers and acquisitions in a short time, resulting in incomplete analysis or omissions. If the selected time is too long, although the impact of the event is considered more comprehensive, interference may be introduced at the same time factors that cause deviations in results.

The financial indicator method refers to selecting suitable financial indicators in a long time span and constructing a model to evaluate the long-term utility of mergers and acquisitions. This income evaluation method based on financial data generally uses indicators such as the profit rate of main business, return on net assets, and earnings per share to investigate the realization of the synergy of corporate mergers and acquisitions. Under normal circumstances, scholars use this method to investigate the long-term wealth effect of mergers and acquisitions. In addition, the mergers and acquisitions in the last year were inspected, focusing on the performance of the target company before and after the merger. The conclusion of the study is that, compared with the average level of the industry, the performance of the target company before and after the merger did not show a significant momentum of improvement [9–14]. However, the conduct of mergers and acquisitions may cause structural adjustments within the group, which in turn will cause some companies to change their business direction after mergers and acquisitions and be classified in another industry [15]. He's research samples were taken from year to year, but similar conclusions were obtained. The significant income of the target and the uncertainty of the income of the master and the merger have caused scholars to shift the focus to the total income of the shareholders of both parties obtained from the merger; that is, to test whether the merger creates value, the income should not be considered from one side, but should be a unified entity after the merger. The research objective is to study whether mergers and acquisitions can produce synergistic effects [16]. Checking the M&A transactions during the years and calculating the total wealth change, it is found that the merger results of the target company and the master merged company are positive in all periods, and the synergy gains are relatively stable within the range of decades. In addition, by studying the performance of the largest merger and acquisition activity that occurred in the United States during the year, the company's performance was measured by the ratio of cash flow to sales, the rate of change in the number of employees, the asset turnover rate, and the pension expenditure of individual employees. The results showed that corporate performance improvement from mergers and acquisitions comes from asset management rather than labor expenditures, and merger integration may help increase the overall value of mergers and acquisitions. In addition, we reached a similar conclusion that the total return is mostly positive, and the synergy seems to play a major driving factor in mergers and acquisitions and takeover activities, and mergers and acquisitions tend to create value. By comparing the cash flow income of the M&A company before and after the merger, it is believed that the M&A company has improved the cash flow income through M&A, and thus it is determined that the M&A company has achieved synergies. He studied the mergers and acquisitions of companies in various industries during the years, and finally concluded that the positive wealth effect obtained by mergers and acquisitions comes from the synergy effect. Since financial indicators have made up for the shortcomings of the capital market to a certain extent, scholars have been currently using this method for analysis [17–21]. Sarala *R* M et al. compared the four accounting indicators before and after the reorganization of the company. The results showed that the earnings per share, return on net assets, and investment income of the sample companies in the year of the reorganization increased compared with the year before the reorganization. The debt ratio has declined, and the magnitude of the change is related to the reorganization method and the relationship between the parties involved in the reorganization [22]. Taking M&A events in the past years as a sample, Feng Genfu selected four indicators of main business income: total assets, earnings per share, net profit total assets, and return on net assets to build a model to measure performance changes before and after the merger. Ge *X* et al. believes that the performance of the listed companies' mergers and acquisitions showed an upward trend and then a downward trend; in different periods before and after the merger, the performance of different types of mergers and acquisitions was inconsistent, and the merger and acquisition integration of listed companies was not successful [23]. Xie *E* et al. believes that the performance of horizontal mergers is significantly better than vertical mergers and hybrid mergers [24].

The advantage of this research method is that it can use a longer accounting period to investigate the realization of mergers and acquisitions synergy. This is more in line with reality and compensates for the shortcomings of the capital market to a certain extent, but at the same time, it will inevitably introduce some irrelevant externalities [25]. Disturbing factors affect the accuracy of the results. In addition, because the data used is historical data, the results produced have limited ability to reflect the current and future expected conditions of the enterprise. In addition, under the current system, financial information of many listed companies is false to varying degrees, and accounting profit indicators are often manipulated. Therefore, the results obtained from financial indicators have certain limitations.

## 2. Fuzzy Comprehensive Evaluation Model of Merger Synergy Based on Transfer Learning Neural Network

### 2.1. Evaluation Ideas of Transfer Learning Algorithm

**Figure 1:**
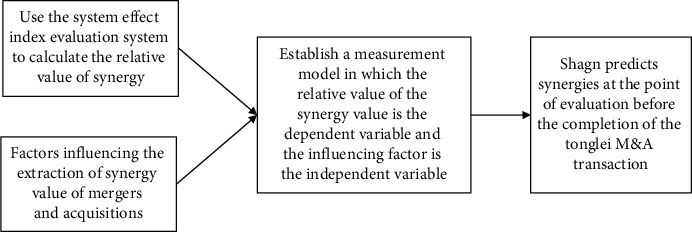
Schematic diagram of setting up the synergy value model of mergers and acquisitions.

It is worth noting that various synergies can ultimately be reflected in the changes in financial data after mergers and acquisitions. Therefore, we can evaluate the synergies obtained by mergers and acquisitions through changes in financial indicators before and after mergers. In the first year. after the merger, some financial indicators of the master merged company showed a small increase, but there was no obvious change. In the second year, after the merger, most of the financial indicators of the merging company showed significant growth, and there was no significant change in the third year after the merger and the second year after the merger. Based on the above research, this article believes that in the year of the merger, the main merger company paid more M&A costs and did not integrate the target company, so no synergy effect occurred or the synergy effect was negative. In the first year after the merger, the main merger company completed the transaction part of the integration of the target company, so the overall synergy of the merger and acquisition began to appear. The integration of most companies was completed in the second year after the merger. The existing literature indicated that the company should basically complete the merger and acquisition within one to two years after the merger. The integration is still not completed in the second year, and the integration effects of these companies in the third and fourth years after the merger will not be very good, and the longer the merger integration time, the higher the merger cost and the greater the merger risk. Therefore, this article believes that the synergy effect reached the highest value after the completion of the integration in the second year after the merger. The synergy effect is not obvious when the integration has been completed in the third year after the merger, and the company resumes normal growth, and the third year after the merger or even longer after the merger, the synergy effect cannot be accurately evaluated due to the long time since the merger and acquisition. Therefore, this article uses the financial data of the master merging company in the year before the merger and the financial data of the master merging company in the second year after the completion of the merger to compare the synergy effect of the merger. It truly reflects the financial status after the merger, and the data from the previous year of the merger transaction can better reflect the operating status before the merger. The merged company should have basically completed the integration in the second year after the merger, and the synergy effect of the master merged company has reached the highest value. Moreover, the financial data at this time excluding the interference of integration and other factors can better reflect the development status of the enterprise after the merger, and the synergy obtained by the main merger enterprise can be more accurately reflected by the comparison with the financial data of the year before the merger.  Figure 1 is a schematic diagram of the M&A synergy value model.

This article sets up the evaluation model for the synergy effect of mergers and acquisitions in two steps: first, this article uses the change rate of 11 financial indicators that reflect the changes in the performance of the main merger before and after the merger to establish an evaluation index system for the synergy of mergers and acquisitions and determine each index. Attributes: in the second step, in order to more objectively evaluate the synergy of mergers and acquisitions, this paper uses transfer learning to find the principal components that can make a comprehensive evaluation of the synergy and determine the weight of each principal component.

### 2.2. Establish an Evaluation Index System under the Transfer Learning Algorithm

This article mainly measures the synergy obtained by the changes in the performance of the main merger before and after the merger, and the change rate of the financial indicators of the enterprise is an important indicator of performance evaluation. Therefore, based on the principles of establishing the indicator system and from the perspective of accurately evaluating synergies, this paper analyzes and screens the financial indicators for evaluating synergies one by one and derives five first-level financial indicators that reflect changes in corporate performance and 11 secondary financial indicators. Based on the change rates of these 11 financial indicators, a synergy evaluation indicator system for mergers and acquisitions was established, and the attributes of each secondary financial indicator's contribution to the synergy were determined. At the same time, each financial indicator was coded. The evaluation indicator system is shown in Table 1.

According to the above, the change rate of financial indicators in this article is the ratio of the financial indicator of the main merged company in the second year after the merger minus the balance of the indicator in the year before the merger and the absolute value of the indicator in the year before the merger. It reflects the changes in corporate performance brought about by mergers and acquisitions. It is worth noting that most companies now face the problem of excessively high debt ratios, so we assume here that the change rate of the quick ratio is a positive indicator, and the change rate of the asset-liability ratio is a negative indicator.

### 2.3. Use Transfer Learning to Find Principal Components and Determine Index Weights

This paper studies the financial indicators that reflect the synergy effect after mergers and acquisitions and obtains the change rate of several financial indicators to measure the size of the synergy effect after mergers and acquisitions. However, we have noticed that there are several problems when using these financial index change rates to evaluate synergy. Importantly, for the accuracy of evaluation, they need to be transformed into comprehensive evaluation indicators that are not related to each other. Third, when evaluating the synergy effect after mergers and acquisitions, the importance of each evaluation index is different. To accurately evaluate the synergy effect, it is necessary to objectively determine the weight of each index.

Based on the above analysis, this paper considers the application of transfer learning to reduce the dimensions of the evaluation index of merger synergy, remove the interference of related factors, and determine the weight of the evaluation index. As a multivariate analysis method, transfer learning is widely used in the comprehensive evaluation of multiple indicators, especially when determining the weight of indicators, transfer learning avoids the subjectivity of previous methods such as expert evaluation.

The migration learning steps are as follows:

  The first step is to standardize the original data and calculate the correlation coefficient matrix,
  The second step is to find the eigenvalues and eigenvectors of the correlation matrix and to get the eigenvalue, eigenvalue contribution rate, and cumulative contribution rate.
  The third step is to select the principal components. The cumulative contribution rate of the selected principal components can reach 80% or more.
  The fourth step is to calculate the principal component load matrix and explain the economic significance of each principal component based on the principal component load matrix.
  The fifth step is to construct a comprehensive score function for synergy

Assuming that *n* principal components are selected, the comprehensive scoring function formula (1) of the synergy of mergers and acquisitions of each enterprise is

(1) $$\begin{array}{c} Y=a_{i1}Y_{i1}+a_{i2}Y_{i2}+\ldots +a_{in}Y_{in}, \end{array}$$

where *Y* is the synergy score of the *i*th company M&A, *a*_*ij*_ is the variance contribution rate of the *j*th principal component index of the *i*th company, and *Y*_*ij*_ is the score of the *j*th principal component index of the *i*th company.

### 2.4. The Process of Establishing a Fuzzy Comprehensive Evaluation Model for the Synergy of Mergers and Acquisitions Based on the Transfer Learning Neural Network

In many cases, it is difficult to establish a linear assumption about the relationship between the synergy value of M&A and its influencing factors. Various influencing factors may affect the implementation of synergy value of M&A through different types of nonlinear ways. Although we can set the nonlinear function form between the influencing factor variable and the synergy value based on the past evaluation experience, the relationship between the artificial preset variable inevitably leads to the setting error of the model, thus making the established model unable to accurately reflect the effect of influencing factors on the synergistic value of mergers and acquisitions, thereby reducing the ability to predict and assess the synergistic value of mergers and acquisitions.

An artificial neural network is a mathematical model that uses a structure similar to the prominent links of the brain's nerves for processing. Usually, there is a nonlinear relationship between input and output values. First, a neural network with a certain number of neurons is established according to the input variable dimension and a certain learning criteria is set to establish the corresponding nonlinear model, then aiming to continuously reduce the error between the network output value and the target value. According to the input value and output value of the sample, the initial setting of the network is continuously modified to learn and train. Finally, the eigenvalues are inputed, reflecting the researched problem into the neural network to get the research result, that is, evaluating the actual problem through the stored information. In addition, the way the artificial neural network obtains the weights based on the actual training methods of the samples avoids the subjective factors and uncertainties of artificially designing the weights, thereby improving the accuracy of the evaluation.

**Figure 2:**
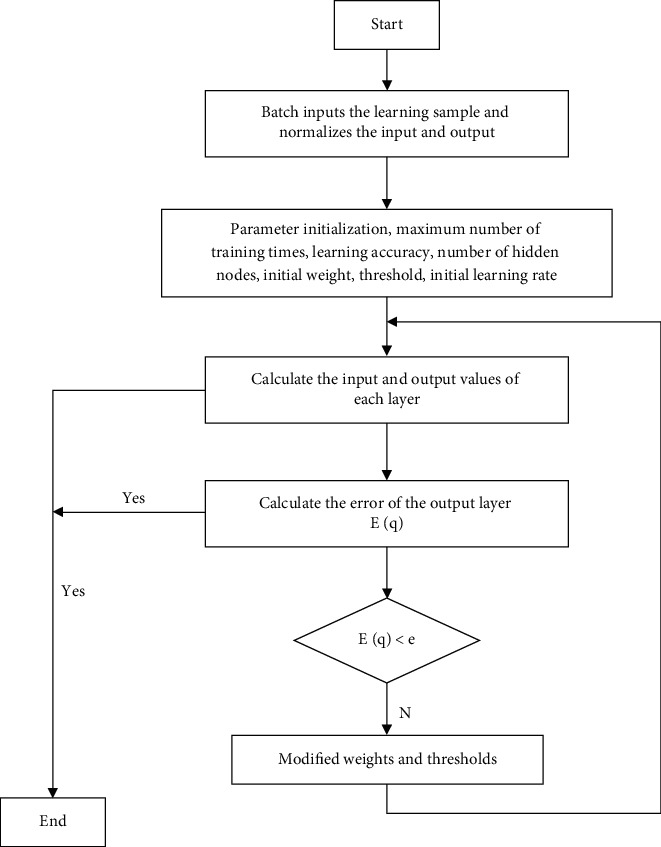
The calculation process diagram of the transfer learning neural network.

**Figure 3:**
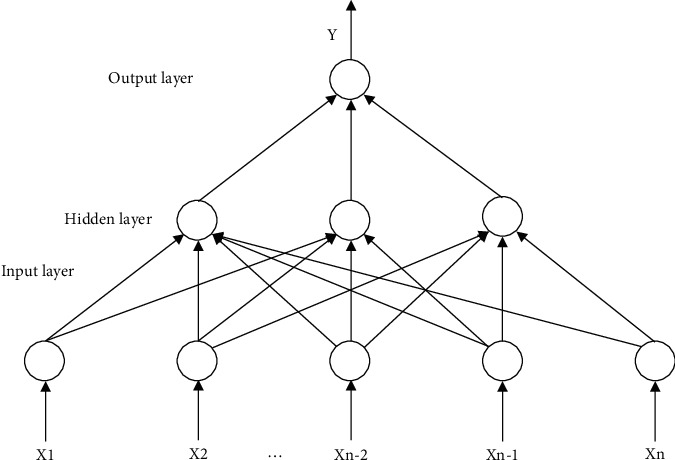
Neural network neuron conduction process diagram.

Among the various types of neural networks, the neural network, that is, the backpropagation network, is the most widely used and successful network form, and it is the essence of the artificial neural network. Therefore, this article also tries to use the neural network to fit the highly nonlinear functional relationship between the influencing factors and the synergistic effect. As a multilayer hope-feeding neural network, the transfer function between the network neurons usually adopts a differentiable monotonic increasing function, such as a logarithmic or tangent function, to achieve any nonlinear mapping from input to output. A typical network structure has an input layer, a single hidden layer, and an output layer. The network structure is shown in the figure. When the forward propagation input information passes from the input layer to the output layer after being processed by the hidden layer, the state of the neurons in each layer only affects the state of the neurons in the next layer. If the desired output cannot be obtained in the input layer, it will switch to backpropagation and return the error signal along the original neuron connection path. During the return process, the weight of the neuron connection of each layer will be modified one by one. This process is iterated continuously, and finally the signal error is within the allowable range, and the actual output of the network is close to the expected output. The process diagrams are shown in Figures 2 and 3:

K. Funahashi's research has proved that a neuron network with a single hidden layer can represent any continuous function with arbitrary precision; and others theoretically prove that a neuron network can approach a large class of functions arbitrarily and can reveal what is in the sample data. It contains the nonlinear relationship. Therefore, the neural network has strong classification capabilities. The three-layer network can achieve convex continuous domain judgments at any given accuracy and can pass various financial and corporate management information that can be known before the conclusion of the merger and acquisition transaction. The specific observation value is used to predict the synergistic value of mergers and acquisitions. If the dependent variable is the synergistic effect of mergers and acquisitions, the independent variable is certain attribute characteristics that affect both companies as shown in the following formula:

(2) $$\begin{array}{c} Y=f\left(x\right);X=\left(x_{1},x_{2},\ldots ,x_{n}\right), \end{array}$$

where *x*_1_, *x*_2_,…, *x*_*n*_ indicate the influencing factors that may be related to the synergy of mergers and acquisitions. In the typical three-layer feedforward neural network structure as shown in the figure, the input vector of the network is the variable data of the factors that affect the synergy effect of mergers and acquisitions that we need to consider, and the output vector of the network is the comprehensive score of the synergy effect of mergers and acquisitions. Through the standardized processing of the data, it is transformed into a number in between. The output layer of the network has 1 node, and the comprehensive score of the same effect is also represented by a numerical value in between. The Kolmogorov theorem points out a formula with reference to the number of neurons in the hidden layer, $n_{1}=\sqrt{n+m}+a$. Among them, there is the number of output neurons, the number of input neurons, and a constant in between. According to this theorem, the reasonable number of hidden layer neurons should be in between. The sample population is then divided into two parts, each accounting for 2/3 and 1/3 of the population sample, which are used as training samples and testing samples, respectively. Since the number of neurons in the hidden layer directly affects the nonlinear prediction performance of the network, the author has repeatedly tried to train and predict the network with different numbers of hidden layer neurons and choose the ideal hidden layer neuron according to the pros and cons of the prediction performance. For a neural network with the number of elements, the test samples to be substituted into different neural network models to predict the comprehensive score of the synergy effect are used, and the results are compared with the actual value to check the predictive ability of the model. The index used to predict the accuracy is the root mean square error as shown in the following formula:

(3) $$\begin{array}{c} \text{Root mean square error}=\sqrt{\frac{1}{J}\sum_{j}^{J}{\left({\hat{F}}_{j}-F_{j}\right)}^{2}.} \end{array}$$

In the above formula, *j* represents the *j*th sample, and there are a total of *J* M&A transaction samples. $\hat{F}$ is the predicted synergy score, and *F*_*j*_ is the actual score obtained by the principal component analysis.

Assume that the sample classification data are divided into two populations A and B, and the output of the *m* hidden layer neurons of a certain network is *X*^*H*^=[*x*_1_^*H*^, ..., *x*_*m*_^*H*^]. Then, the linear classifier comprised of the optimal output layer can be obtained by

(4) $$\begin{array}{c} y=V^{\prime }X^{H}. \end{array}$$

The optimal weight satisfies the following formula:

(5) $$\begin{array}{c} V^{*}=\arg\;\max J^{O}\left(V\right)=\arg\;\max\frac{{\left({\bar{y}}_{A}-{\bar{y}}_{B}\right)}^{2}}{\sum_{i\in A}{\left(y_{i}-{\bar{y}}_{A}\right)}^{2}+\sum_{j\in B}{\left(y_{i}-{\bar{y}}_{B}\right)}^{2}}. \end{array}$$

The weight calculation steps are as follows:

(6) $$\begin{array}{c} S_{V}=S_{A}+S_{B}=\sum_{X\in A}\left(X^{H}-{\bar{X}}_{A}^{H}\right){\left(X^{H}-{\bar{X}}_{A}^{H}\right)}^{\prime }+\sum_{X\in B}\left(X^{H}-{\bar{X}}_{B}^{H}\right){\left(X^{H}-{\bar{X}}_{B}^{H}\right)}^{\prime },\\ V=S_{V}^{-1}\left({\bar{X}}_{A}^{H}-{\bar{X}}_{B}^{H}\right). \end{array}$$

The threshold can be obtained from the following formula:

(7) $$\begin{array}{c} a=\frac{{\bar{y}}_{A}-{\bar{y}}_{B}}{2}. \end{array}$$

Both the neuron population *S*^(*t*)^ and the hidden layer population *P*^(*t*)^ adopt two-dimensional coding, which are defined as the following formulas:

(8) $$\begin{array}{c} S^{\left(t\right)}=\left\{w_{1}^{\left(t\right)},w_{2}^{\left(t\right)},\ldots ,w_{qK}^{\left(t\right)}\right\}=\left[\begin{array}{cccc} W_{1,1} & \cdots & W_{1,n} & b_{1}\\ \vdots & \ddots & \vdots & \vdots \\ W_{Kq,1} & \cdots & W_{Kq,1} & b_{Kq} \end{array}\right], \end{array}$$

(9) $$\begin{array}{c} P^{\left(t\right)}=\left\{c_{1}^{\left(t\right)},c_{2}^{\left(t\right)},\ldots ,c_{K}^{\left(t\right)}\right\}=\left[\begin{array}{ccc} c_{1,1} & \cdots & c_{1,Kq}\\ \vdots & \ddots & \vdots \\ c_{K,1} & \cdots & c_{K,Kq} \end{array}\right], \end{array}$$

where *t* and *n* are the evolutionary algebra and the number of neurons in the input layer, respectively. The settable model parameters *q* and *K* are the number of hidden layer neurons and the hidden layer population size, respectively, and the neuron population size is Kq to ensure that there are a sufficient number of candidate nodes. *w*_*ij*_, *b*_*i*_, *c*_*kj*_ is the threshold of the real number weight domain and the binary variable, respectively. When initializing *S*^(0)^, all weights and thresholds are normally distributed random numbers with zero mean and finite variance, and the range of values is not limited during the evolution process. *P*^(0)^ is a random 0-1 number and satisfies ∑_*j*=1_^*Kq*^*c*_*ij*_=*q*. It remains unchanged during the evolution process, that is, the network scale remains unchanged.

In addition, different types of misjudgments in practical applications often bring different misjudgment costs. These indicators can also be substituted into the fitness function to bring greater penalties for misjudgments that decision makers do not want and guide the optimization search direction. Assuming that the training target classification result is *T*, the two types of misjudgment costs are *r*_*A*_ and *r*_*B*_, respectively. The fitness of this paper is calculated as follows:

(10) $$\begin{array}{c} j_{i}^{H}=\left\{\begin{array}{c} r_{A},T_{i}\neq \text{yi and }i\in A,\\ r_{B},T_{i}\neq \text{yi and }i\in B,\\ 0,\text{ else}. \end{array}\right) \end{array}$$

(11) $$\begin{array}{c} J^{H}=\frac{\left\|D_{V}\right\|\sum_{i\in D_{T}}j_{i}^{H}+\left\|D_{T}\right\|\sum_{i\in D_{V}}j_{i}^{H}}{\left\|D_{V}\right\|+\left\|D_{T}\right\|}. \end{array}$$

## 3. Results and Discussion

### 3.1. Synergy Score of Each Merger Event

The research results from Table 2 show that the comprehensive relative value of the synergy of many companies' mergers and acquisitions is negative, but it does not mean that the synergy of the mergers and acquisitions obtained by these companies is negative. The positional relationship of the average level of synergy is obtained. Therefore, these relative values must be further explained.

First of all, the change rate of various financial indicators after the acquisition of Shenshenbao by agricultural products shows that, except for the change rate of the main business profit rate and the change rate of the asset-liability ratio, the change rate of other financial indicators has appeared more obvious. Therefore, we conclude that the synergy effect of the agricultural product acquisition Shenshenbao may be negative, and the comprehensive score of the acquisition event is −0.423. Next, it is necessary to verify whether the synergy effects of mergers and acquisitions with a comprehensive score lower than −0.423 are all negative. The calculation result of the comprehensive score of the merger event shows that the comprehensive score is slightly lower than −0.423 for the Hengyang economic development of China National Travel Service. The rate of change of various financial indicators shows that all the original data are negative. From this, it can be judged that China National Travel Service's merger and acquisition of Hengyang's economic development has achieved a negative synergy effect. In conclusion, although we have noticed that not all mergers and acquisitions have a synergy score of less than −0.423, the rate of change of various financial indicators is negative. Transforming multiple related variables into several unrelated comprehensive variables through dimensionality reduction is studied, and the importance of each comprehensive variable is different. This will happen even if the rate of change of various financial indicators is not all negative. However, the comprehensive score of M&A synergy is lower than the result of −0.423.

Second, we will study the mergers and acquisitions with a comprehensive score higher than −0.423 for the synergy of mergers and acquisitions. The calculation results of the comprehensive score of mergers and acquisitions show that the mergers and acquisitions with a comprehensive score of slightly higher than −0.423 are the acquisition of Chaodong and Sanai by Conch Cement, Fu M&A Shanghai Coking, and Shanghai SIPG Group M&A *G* SIPG; according to the change rate of various financial indicators before and after the merger of these three main merged companies, at least 7 of each company's various indicators and the rate of change of other financial indicators are positive. There was no obvious negative growth. Among them, the change rate of 8 financial indicators before and after the merger of SIPG Group showed a relatively obvious positive growth, and the other 3 indicators showed only a slight negative growth. At this time, the comprehensive score of the merger event was −0.387, so we can get a preliminary result. When the comprehensive score of synergy of mergers and acquisitions is higher than −0.387, the company may obtain a positive synergy. In this regard, we can verify this conclusion through the research results of other researchers. He Ke studied various financial indicators after the acquisition of Hualian Commercial Building by No.1 Department Store and concluded that the No.1 Department Store has achieved synergistic effects. In this paper, the synergy score of the merger and acquisition event is −0.281, which is higher than −0.387.

Finally, based on the above analysis, this paper can draw the conclusion that when the comprehensive M&A synergy score is higher than −0.387, the company can obtain a positive synergy, and the M&A synergy with a comprehensive score lower than −0.423 is negative. According to this conclusion, companies can evaluate the size of their own acquisition synergy. In the study of this paper, the relative value of the merger synergy calculated by the synergy effect postevaluation model is used as the output target value of the BP neural network prediction model. Since the predicted relative value of merger synergy is of strong comparative significance, the predicted value of the merger synergy is relatively significant. Comparing the relative value of synergy with other companies, the company can predict the degree of synergy in mergers and acquisitions in advance, and it can also provide a basis for the selection of target companies and the determination of the price of mergers and acquisitions.

In addition, through the above conclusions, we noticed that in all the research samples, only 8 M&A events have a comprehensive M&A synergy score lower than −0.387, and the remaining 20 M&A events have a M&A synergy score higher than −0.387, that is, 70% of the companies achieved synergies after mergers and acquisitions, which proves that the master merged company can obtain the synergy effect of mergers and acquisitions. It denies the view that it is difficult for the master merged company to obtain synergies in mergers and acquisitions compared to the target company.

### 3.2. Estimation Results of the Fuzzy Comprehensive Evaluation Model of Merger Synergy Based on Transfer Learning Neural Network

**Figure 4:**
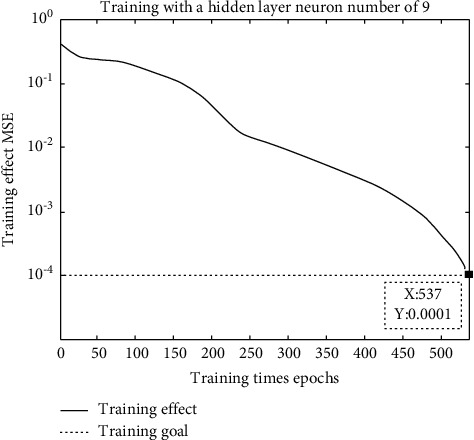
Training with 9 neurons in the hidden layer.

Since a single hidden layer network can approximate an arbitrary continuous nonlinear function, we use a single hidden layer network to predict the value of mergers and acquisitions. Our input sample is a dimensional input variable vector, that is, the proportion of cash payments, whether related transactions, the relative scale of mergers and acquisitions, the proportion of state-owned shares, premerger performance, premerger scale growth, and equity concentration. Therefore, the input layer is a neuron. The network has only one output variable, that is, the comprehensive premerger score we need to predict, so the output layer has only one neuron. The choice of the number of hidden layer nodes is a complicated problem, which is directly related to the number of input and output layer nodes. According to the aforementioned empirical formula for the number of hidden layer nodes, the number of hidden layer nodes may have the best value in the range. For the selected sample data, the “try” method is used. First, a relatively small number of hidden layer nodes is set for training. If there is no convergence within the specified number of training times, training is stopped; then, the number of hidden layer nodes is gradually increased, and training is restarted. Specifically, we have successively trained the number of hidden layer nodes in the same situation; the accuracy of the neural network can accurately test the nodes. If the test fails within the specified number of times, the test converges and stops. Before the training of the network, we normalized the training samples and prediction samples and processed the data into intervals (in-between data). After that, we trained and predicted the network. Aiming at such networks with different structures, this paper sets the transfer function of the hidden layer neurons as the type tangent function, and the transfer function of the output layer neurons as the type logarithmic function and uses this function as the training function. The gradient descent method is used for learning, and the learning rate is adaptive. Through comparison, it is found that the network has reached the training goal after the second training, and the convergence speed is faster as shown in Figure 4.

**Figure 5:**
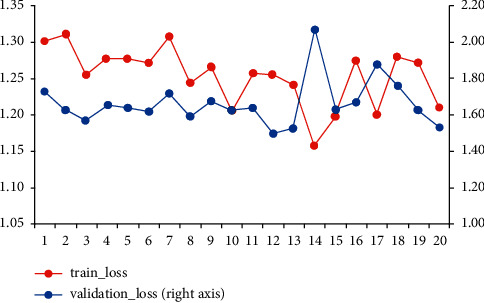
Loss change graph of the generalized neural network.

Combined with the comparison of training times, this paper adopts a structured network as a feasible neural network model that can be used to evaluate the possible merger effects created by mergers and acquisitions at the point of evaluation before the completion of mergers and acquisitions.  Figure 5 shows the loss value change graph. It can be seen from the change graph that the optimal training round is 12 rounds, the final model batch number is 280, the learning rate is 0.1, and *λ* is 1.3.

**Figure 6:**
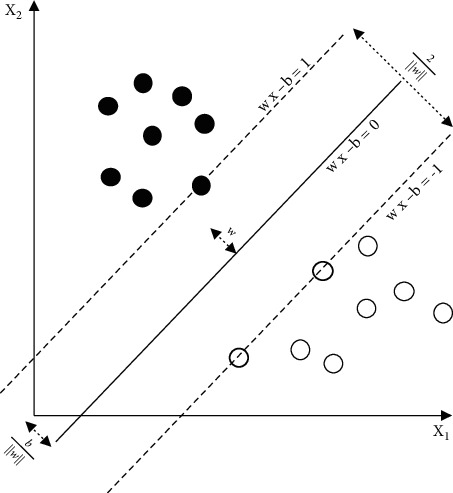
Evaluation of the dataset by the transfer learning neural network.

The transfer learning neural network also requires a rigorous evaluation of the difficult dataset in the weak label scenario, as shown in Figure 6 and shows that their method is better than the existing technology at the time by 12% in accuracy of each category. A comparable accuracy was maintained.

**Figure 7:**
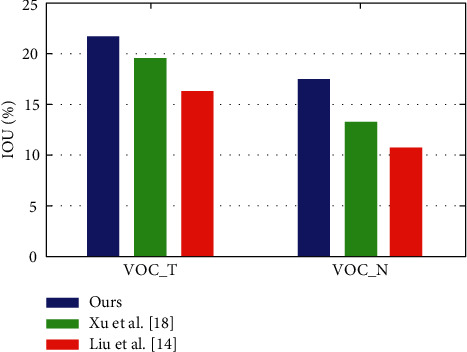
Comparison of the label accuracy of the transfer learning neural network and other algorithms in a weak interference environment.

**Figure 8:**
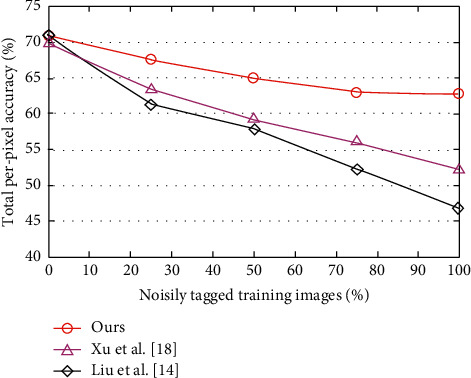
Comparison of prediction accuracy between transfer learning neural network and other algorithms.

However, when more and more training images are damaged by noisy labels, this advantage quickly disappears. When all training images contain noisy labels, our method has an improved 13.6% advantage over [17]. Note that all three methods have exactly the same model input (superpixel-segmentation and representation are the same); their representation models are also similar (linear models). Therefore, these results provide clear evidence that our method is more robust to label noise due to its clear and direct noise reduction model. As shown in Figure 7, the transfer learning neural network model can also be improved by predicting accuracy. Good results are obtained, as shown in Figure 8.

**Figure 9:**
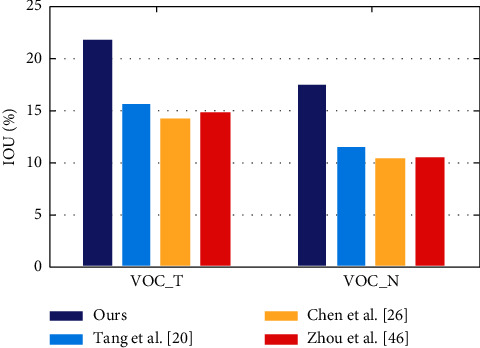
Comparison of the label accuracy of the transfer learning neural network and other algorithms in a strong interference environment.

For a fair comparison, the picture shows the accuracy of each model on the label in a strong interference environment. It can be seen that no matter what features are used, the transfer learning neural network method is significantly better than the other methods. This shows that the improvement comes mainly from the model itself, not the function used. Note that given the very noisy tags (e.g., 75%), the effect gap is particularly large (see Figure 9).

## 4. Conclusion

In a sense, the accuracy of predicting the synergy of mergers and acquisitions plays a crucial role in the success or failure of mergers and acquisitions. It is an important basis for selecting target companies and determining their prices. Therefore, we can say that the accurate estimation of the synergies of mergers and acquisitions can effectively resolve the risks of mergers and acquisitions and improve the success rate of mergers and acquisitions. Different from previous studies, this article does not use traditional methods such as cash flow and stock return changes when predicting the synergy of mergers and acquisitions. Instead, it uses neural networks to fit the relationship between the influencing factors of synergy of mergers and acquisitions and the relative value of synergy after mergers and acquisitions. A M&A synergy prediction model is constructed, and the relative value of M&A synergy predicted by this model is compared with the relative value of synergy achieved by other completed M&A companies to predict the results of M&A and reduce blindness in M&A. In addition, the research results show that this neural network model can predict the synergy effect of mergers and acquisitions more accurately. Therefore, the model has good practical significance. In future practice, we only need to input the value of the influencing factors that affect the synergy effect before the merger into the prediction model, and the relative value of the synergy effect after the merger can be obtained by comparing this relative value with the synergy effect achieved by other completed mergers and acquisitions. Value comparison can be used to analyze whether the company can profit from mergers and acquisitions.

## Figures and Tables

**Table 1:** Synergy evaluation index system.

First-level indicator	Secondary indicators	Attributes	Code
Profitability change index	Rate of change of return on equity	Positive	*X*_1_
	Change rate of main business profit margin	Positive	*X*_2_

Growth ability change index	Rate of change of net asset growth rate	Positive	*X*_3_
	Change rate of main business income growth rate	Positive	*X*_4_

Cost control ability change index	Rate of change in management expense margin	Positive	*X*_5_
	Rate of change in operating expense margin	Positive	*X*_6_

Asset management level change indicators	Change rate of inventory turnover	Positive	*X*_7_
	Rate of change of liquid asset turnover	Positive	*X*_8_
	Change rate of total asset turnover	Positive	*X*_9_

Change in solvency index	Rate of change of quick ratio	Positive	*X*_10_
	Rate of change of the debt-to-asset ratio	Inverse	*X*_11_

**Table 2:** Comprehensive score.

Serial number	1	2	3	4	5	6	7	8
Score	0.596	0.557	2.881	1.478	0.164	−0.306	−0.024	−0.298
Serial number	9	10	11	12	13	14	15	16
Score	−0.084	0.560	−0.477	−0.423	0.158	−0.281	−0.495	−0.387
Serial number	17	18	19	20	21	22	23	24
Score	−0.409	0.018	−0.869	1.044	−1.246	−0.768	−0.394	0.253
Serial number	25	26	27					
Score	−0.155	0.121	−0.210					

## Data Availability

The data used to support the findings of this study are available from the corresponding author upon request.
